# Effect of buttress plate in Herscovici type D vertical medial malleolar fractures and peripheral fractures: a retrospective comparative cohort study

**DOI:** 10.1186/s13018-023-03889-0

**Published:** 2023-06-07

**Authors:** Bing Luo, Yuqi Wang, Dewei Wang

**Affiliations:** 1 Weifang Medical University, No. 7166 Baotong West Street, Weicheng District, Weifang, 261053 China; 2 Jinzhou Medical University, Jinzhou, China

**Keywords:** Buttress plates, Herscovici type D, Medial malleolar vertical fractures, Supination-adduction injuries

## Abstract

**Background:**

The purpose of current retrospective study was to review the surgical methods and to evaluate the clinical efficacy of supporting plate for the treatment of vertical medial malleolus fractures on the basis of stable fixation of ipsilateral fibula.

**Methods:**

This retrospective study included a total of 191 patients with vertical medial malleolus fractures. Patients enrolled were divided into simple vertical medial malleolus fractures and complex types of fractures. General demographic information and surgical information, including age, sex, surgical procedure and postoperative complications, were collected. The functional prognosis of patients was evaluated by American Orthopedic Foot and Ankle Society Ankle-Hindfoot Score (AOFAS) and Visual Analog Scale (VAS).

**Result:**

Among patients with simple vertical fractures, the respective incidence of internal fixation failure in screw group, buttress plate group, and screw combined buttress plate fixation group (combined fixation group) was 10/61 (16.4%),1/54 (7.4%) and 1 (1.9%), and the difference was statistically significant (*P* = 0.024). The incidence of abnormal fracture growth and healing in screw group, buttress plate group and combined fixation group was, respectively, 13/61 (21.3%), 6/54 (12.5%) and 2 (3.85%), with statistically significant difference (*P* = 0.019). In the patients with complex types of fractures, after 2 years of postoperative follow-up, the AOFAS score and VAS score of the following subgroups had good results: 91.18 ± 6.05 and 2.18 ± 1.08 in patients with joint surface collapse, and 92.50 ± 4.80 and 2.50 ± 1.29 in patients with tibial fractures, with 100% excellent and good rate.

**Conclusion:**

For simple and complex vertical medial malleolus fractures, buttress plate showed excellent fixation. Despite poor wound healing and extensive soft tissue dissection with this approach, buttress plate may provide a novel insight into medial malleolar fractures, especially for extremely unstable medial malleolar fractures.

## Introduction

Ankle fracture is a common fracture type, which presents in about 10% of all fractures [1, 2]. Ankle fractures can be caused by direct or indirect force that includes rotation, inversion, axial movement, and axial force [3]. The main objective of ankle fracture treatment is to achieve good joint stability, thereby allowing for early functional exercise, promoting fracture healing and preventing the occurrence of osteoarthritis. In recent years, increasing attention has been paid to the medial structure of the ankle. The medial structure of the ankle joint mainly consists of the medial malleolus and the triangular ligament. Due to the special anatomy and injury mechanism of the medial malleolus, the fracture of the medial malleolus has a complicated condition, among which the supination-adduction injuries are even more difficult to treat. Low lateral malleolus fracture or lateral collateral ligament injury can result in vertical split fracture of medial malleolus, and compression fracture of the distal medial articular surface of tibia may occur [4, 5]. This fracture pattern is relatively rare, accounting for 12.2% to 21.1% of ankle fractures [6]. Herscovici et al. proposed a classification that divided medial malleolus fractures into four different types based on X-ray images, and simply and intuitively described the shape of the fracture end, which is a commonly used clinical classification for the evaluation of medial malleolus fractures [7].

The current standard of treatment for medial malleolus fractures is open reduction and internal fixation [8]. There are multiple options available for internal fixation, such as single or bicortical screws, headless screws, half-tension wire, medial malleolus plate internal fixation, etc. [9–12]. However, the best fixation method is still an ongoing debate about the superior technique because of the limited evidence and researches [13]. The analysis of the force of the ankle joint has revealed that the vertical fracture line should bear both the vertical load force and the horizontal force from the body during the ankle joint movement. Such a large shear force prevents conventional Kirschner wires, screws, ordinary plates and other fixation measures from properly neutralizing the shear force, resulting in internal fixation failure, internal fixation fracture or secondary fracture displacement [14]. Herein, the aim of this study was to evaluate the clinical efficacy of Buttress plate for the treatment of vertical medial malleolus fractures on the basis of stable fixation of ipsilateral fibula.

## Materials and methods

### Study population

This study retrospectively analyzed 1053 patients with ankle fractures who were treated in our hospital from January 2014 to May 2019. Inclusion criteria: (1) patients with medial malleolus fracture that was vertically out of shape, with or without articular surface collapse; (2) patients with closed fractures; (3) injury to operation time ≤ 1 week; (4) patients over 16 years old, with a closed epiphyseal line; (5) patients involving in a study of internal fixation of fractures. Exclusion criteria: (1) combination with neurovascular injury and fracture of bones around the foot (such as the talus, with the exception of the tibia and fibula); (2) medial malleolus fractures that could not be classified as Herscovici-type supination-adduction type II or Herscovici type D [15]; (3) isolated medial malleolus fracture; (4) patients who had severe medical diseases, tumors, osteoporosis and other immune diseases; (5) patients who required conservative treatment or transfer; (6) patients who had not completed follow-up for removing the internal fixation device or did not cooperate with the system scoring.

**Fig. 1:**
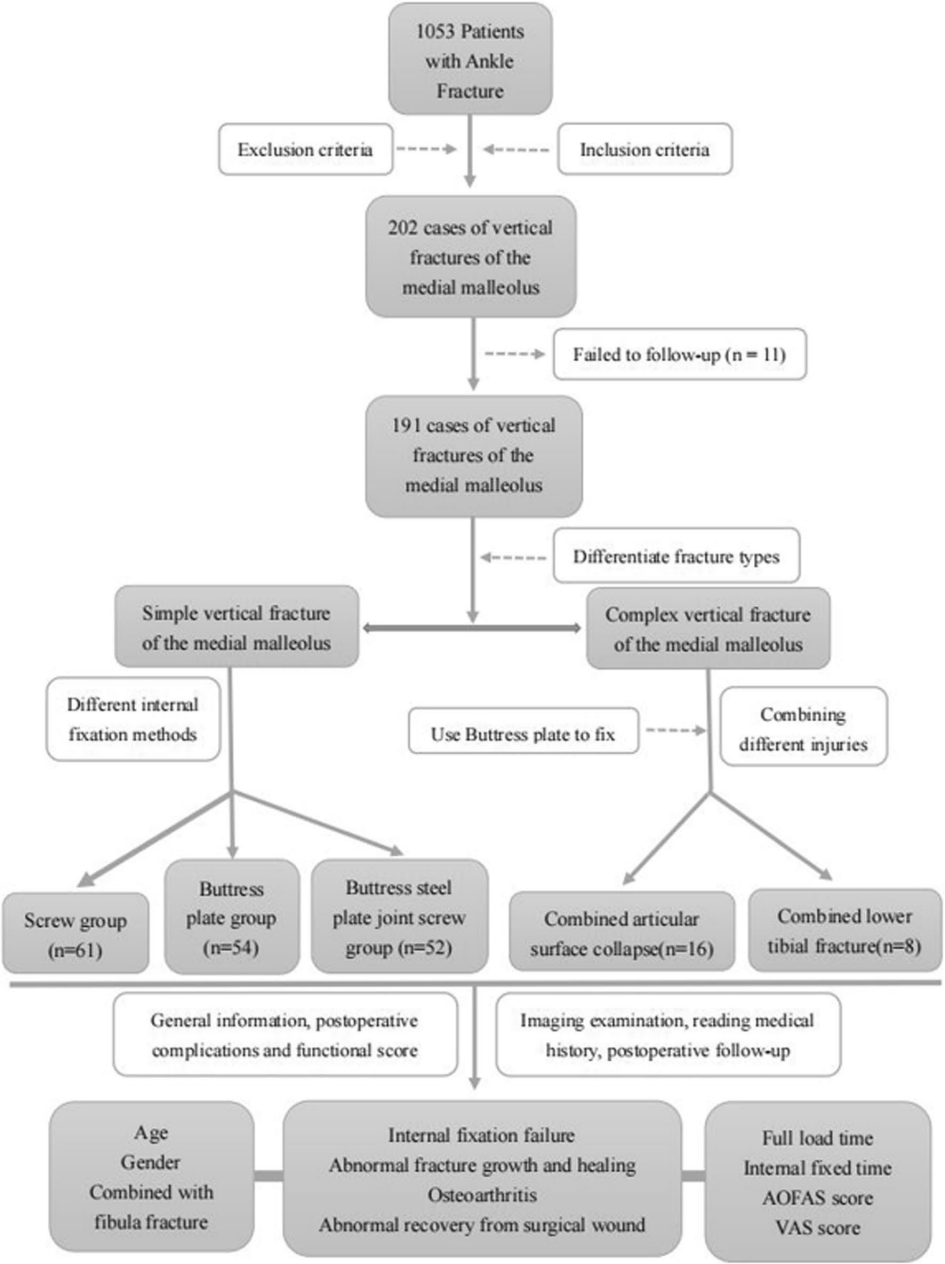
Flow chart of the technical method

**Fig. 2:**
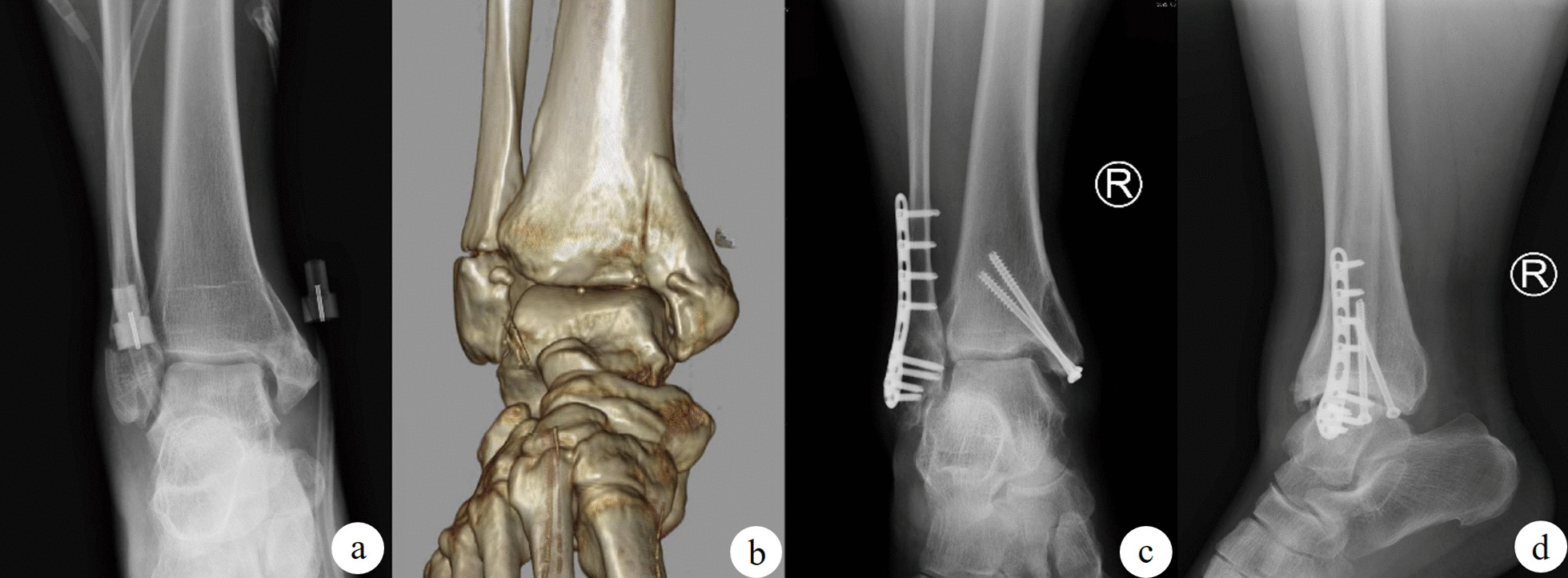
The use of screws to fix medial malleolar fractures is a classic fixation method. **a** The orthographic X-ray images of the fracture; **b** the CT three-dimensional reconstruction image after the fracture; **c, d** the orthographic X-ray images after screw internal fixation

**Fig. 3:**
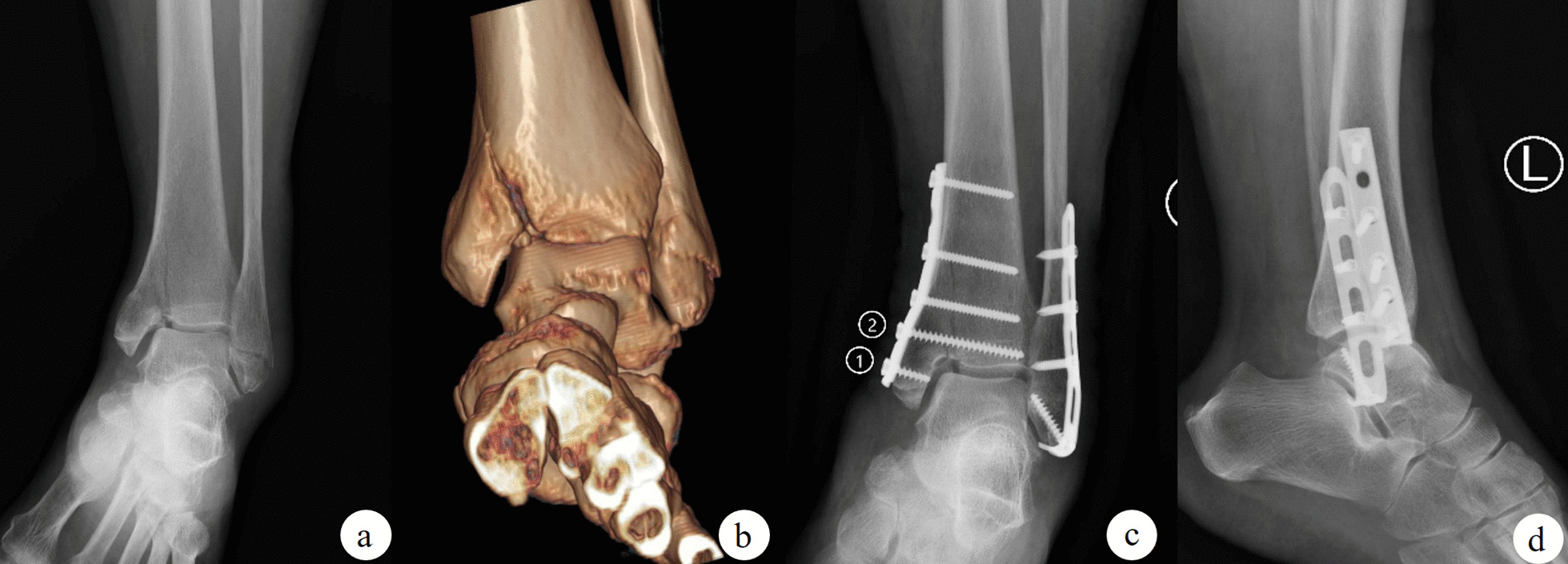
When using the buttress steel plate, the steel plate is prebent into a shape that can be close to the bone surface, and the elasticity and firm structure of the steel plate itself are used to provide fixation, thereby preventing the longitudinal translation of the fracture. However, if only the buttress plate is used for fixation, only one screw **c-2** has the function of fixing the fracture because the most distal screw **c-1** only fixes the tip of the medial malleolus. **a** The orthographic X-ray images of the fracture, **b** the three-dimensional CT reconstruction image after the fracture, and **c, d** the orthographic X-ray images after buttress plate internal fixation

**Fig. 4:**
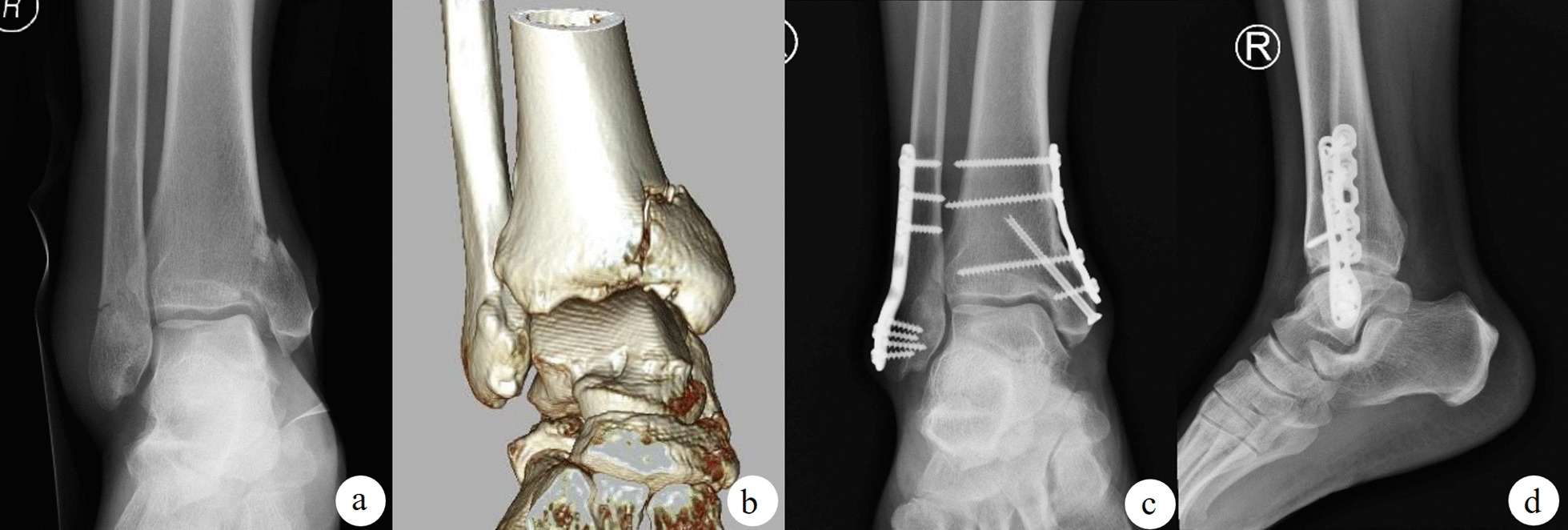
The use of buttress steel plates to assist screw fixation can eliminate the disadvantages of insufficient distal fixation. **a** The orthographic X-ray of the fracture; **b** the three-dimensional CT reconstruction after the fracture, **c, d** the orthographic X-ray of the Buttress plate combined with screw internal fixation

**Fig. 5:**
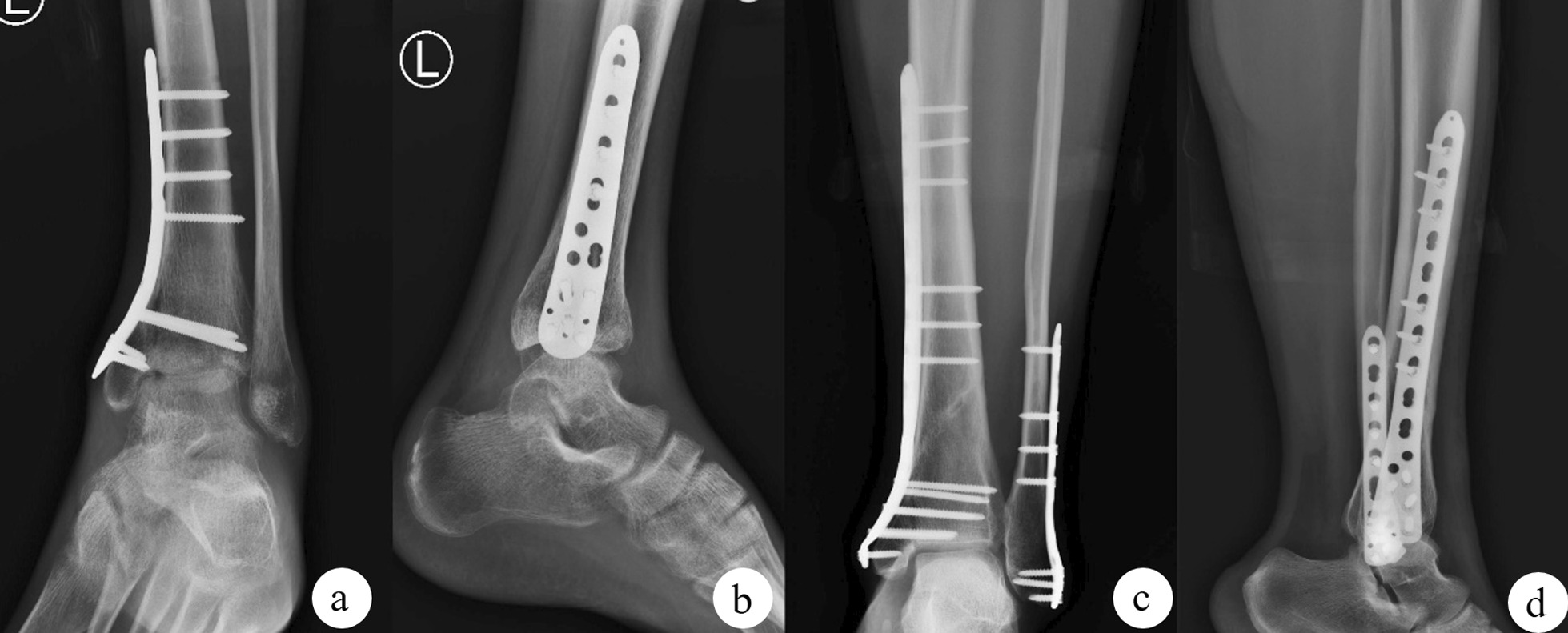
In the supination-adduction injury mode, it is easy to cause displacement of the fracture fragment and collapse of the articular surface (**a**), and other external forces can also cause lower tibial fracture around the fracture line (**c**). The appearance of these two types of fractures increases the difficulty of vertical fractures of the medial malleolus, and a suitable shape of locking plate prebending can be considered a buttress plate to fix the two types of fractures (**b**, **d**)

A total of 202 people met the selection criteria, and finally 191 of them completed the follow-up to remove the internal fixation (Fig. 1). Of these patients, 61 (31.9%) received screw fixation (Fig. 2), 54 (28.3%) received buttress plate internal fixation (Fig. 3), and 52 (27.2%) received buttress plate combined with screw fixation (Fig. 4). Of the remaining 24 patients, 16 (66.7%) patients had articular surface collapse (Fig. 5a), and 8 (33.3%) patients had tibial fractures (Fig. 5c), and the patients were all treated with buttress plate internal fixation (Fig. 5b, d). Patients were divided into simple vertical medial malleolus fractures and complex types of fractures. Patients with simple vertical fractures were then subdivided into screw group, buttress plate group, and screw combined buttress plate fixation group (combined group) according to internal fixation methods for comparison. Patients with complex types of fractures were subdivided into those with combined articular surface collapse and combined lower tibial fractures for comparison. Their surgical records and related imaging examinations were screened and consulted for cases using screw fixation, buttress plate fixation, and buttress plate combined with screw fixation. All procedures are performed by the same surgical team with a senior orthopedic surgeon in a tertiary care hospital. All imaging data in this study were independently reviewed by two musculoskeletal radiologists (not involved in the surgery), and the same reading results were obtained.

This study was approved by the Ethics Committee of our hospital and follows the guidelines of the Declaration of Helsinki.

### Observation indicators

Through reviewing medical records and follow-up statistics, the information including age, sex, fibular fracture, complete weight-bearing and internal fixation time of the research target were reviewed.

We monitored and recorded the surgical complications, such as the internal fixation failure after internal fixation (the internal fixation device was bent or broken, the fracture was displaced again, or the alignment of the fracture ends was poor), the abnormal growth and healing of the fracture (delayed union or nonunion of the fracture, or if there were free bone pieces), comorbid osteoarthritis and osteoporosis, and abnormal surgical wound healing (redness and swelling ≥ 3 days, infection, abnormal discharge, delayed wound healing).

### Postoperative follow-up and functional exercise

The American Orthopedic Foot and Ankle Association Ankle-Hindfoot Score (AOFAS) [16] and the Visual Analog Scale for Pain (VAS) [17] were completed 2 years after the operation. The patients were scheduled a review at 1, 3, 6, and 12 months after surgery. The time to remove the internal fixation, depending on fracture healing, was approximately 1 year postoperatively. A final follow-up visit was completed at 2 years postoperatively. After 4–6 weeks of plaster or brace immobilization postoperatively, functional ankle exercises were performed. The gradual weight-bearing functional exercise was started after 4 weeks, and full weight-bearing functional exercise was started after 8 to 12 weeks according to the fracture healing condition.

### Statistical analysis

All the data in this study were analyzed and graphed using SPSS 25 software (IBM SPSS Inc., Chicago, USA), and GraphPad Prism 8 software (GraphPad Software, LLC, La Jolla, California, USA). The measurement data were expressed by $\overline{x}$ ± *s*, and the analysis of variance and *t* test were used for statistical analysis. The counting data were expressed as percentage, and the Chi-square test and Fisher’s exact test were applied for statistical analysis between groups. Power analysis was performed in SAS (SAS 9.1.3 Help and Documentation, SAS Institute Inc, Cary, NC, USA) to evaluate the sample size. It was determined that a minimum of 38 patients was required to achieve a power of 0.8, using an alpha of 0.05 and assuming a correlation of value 0.5. Data with a 2-tailed *P* value of < 0.05 were considered statistically significant for this analysis.

## Results

### Simple vertical medial malleolus fractures

In the three groups of simple vertical medial malleolar fractures, the data of sex, age and combined fibular fractures of each group were analyzed. And there was no significant difference in age and gender among the three groups (*P* > 0.05).

The incidence of internal fixation failure, abnormal fracture growth and healing, osteoarthritis and osteoporosis in screw group, buttress plate group, and combined fixation group showed significant difference (all *P* < 0.05, Table 1).

**Table 1:** Basic data and complication statistics of patients with simple vertical fracture of the medial malleolus

Index	Screw group (*n* = 61)	Buttress plate group (*n* = 54)	Joint fixed group (*n* = 52)	*P* value
Gender (male/female)	40/21	38/16	35/17	0.858*
Age	51.26 ± 9.38	49.41 ± 7.90	49.64 ± 8.63	0.692^#^
Height (cm)	169.44 ± 6.82	171.89 ± 7.75	170.45 ± 8.01	0.302
Weight (kg)	68.35 ± 7.92	66.70 ± 13.17	66.88 ± 12.88	0.622
Combined fibula fracture (*n*)	46	40	37	0.874*
Internal fixation failure (*n*)	10	4	1	0.024*
Abnormal fracture growth and healing (*n*)	13	6	2	0.019*
Combined with osteoarthritis (*n*)	14	8	2	0.015*
Combined with osteoporosis (*n*)	10	16	2	0.002*
Wound abnormality (*n*)	5	6	6	0.811*

^#^, compared by one-way ANOVA analysis; *, compared by Chi-square test

The respective incidence of internal fixation failure in screw group, buttress plate group, and combined fixation group was 10/61 (16.4%), 1/54 (7.4%) and 1 (1.9%), and the difference was statistically significant (*P* = 0.024).

**Fig. 6:**
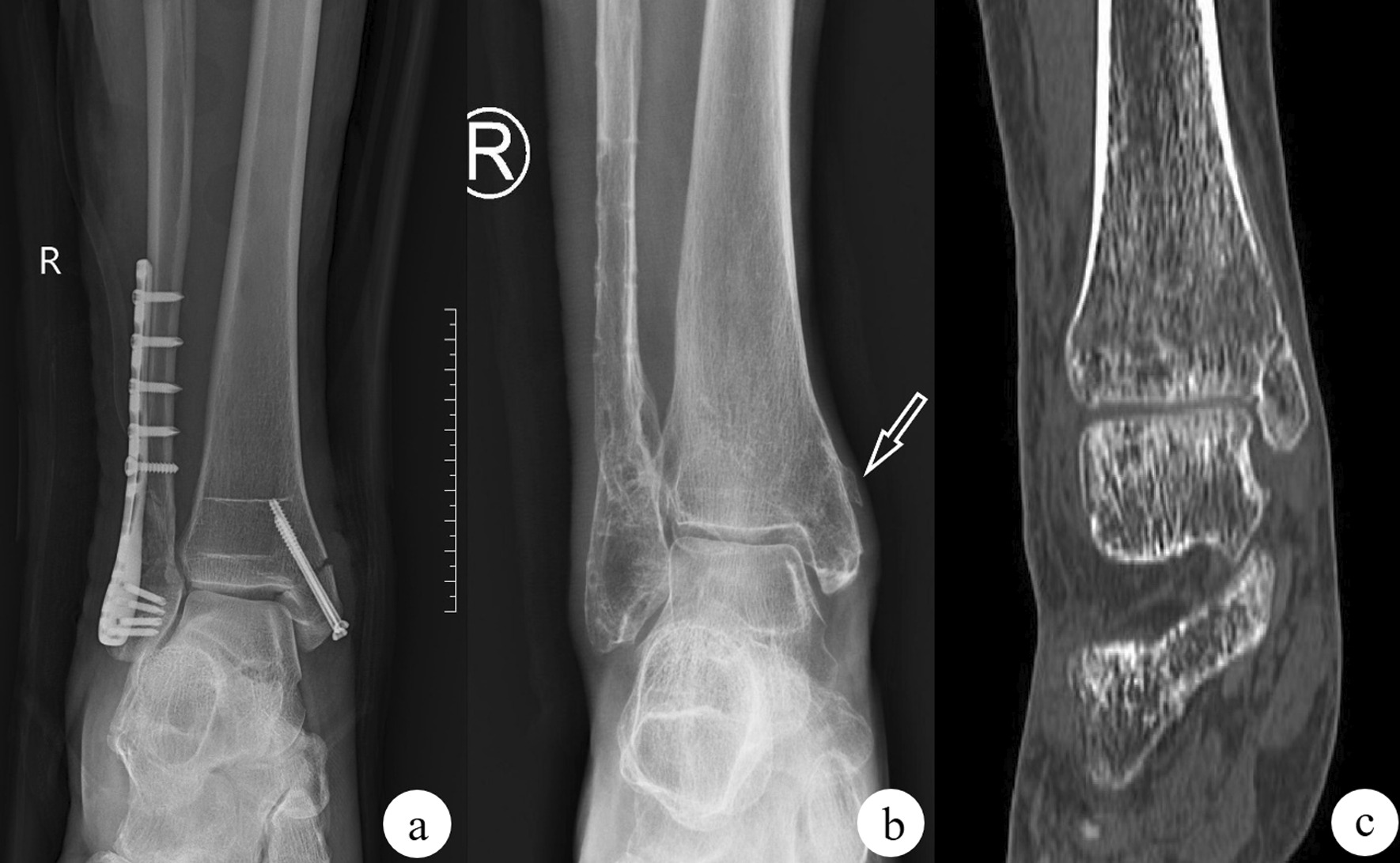
Displacement of the fracture is the key to measuring the success of the internal fixation device. In the screw fixation group, more fractures were re-displaced (**a**), indicating that the screw fixation effect was poor. In addition, screw fixation has a unique X-ray manifestation: the tiny bone piece at the proximal cortex of the fracture line is lifted (**b**, white arrow). In the plate fixation group, there were 16 cases of disuse osteoporosis caused by nonslip plate fixation (**c**)

The incidence of abnormal fracture growth and healing in screw group, buttress plate group, and combined fixation group was respectively 13/61 (21.3%), 6/54 (12.5%) and 2 (3.85%), and the difference was statistically significant (*P* = 0.019). Among the 10 patients in the screw group who had failed fixation, 3 had broken screws and 7 had re-displaced fractures (Fig. 6a). Among the patients with abnormal fracture union in the screw group, 4 suffered from a bulge around the fracture line (Fig. 6b) that was worsened when pressed during follow-up pain.

Disuse osteoporosis was observed in 16 patients with buttress plate fixation (Fig. 6c). The incidence was significantly higher than that of the other two groups (*P* = 0.002) (Table 1).

**Fig. 7:**
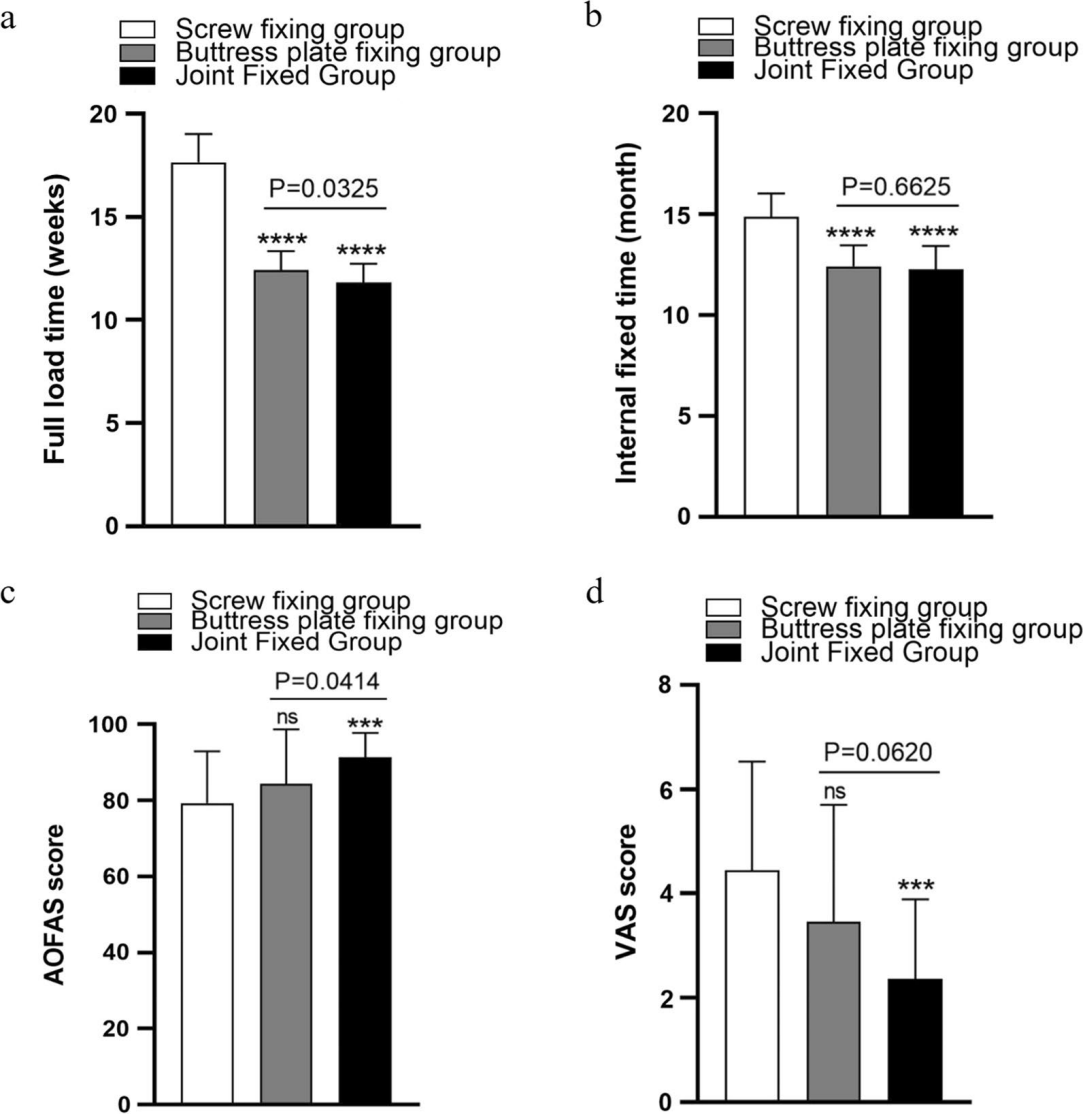
Comparison of the complete weight-bearing time (weeks), internal fixation time (months), AOFAS score and VAS score of the three groups of internal fixation methods for simple medial malleolar fractures. “ns” indicates no statistically significant difference compared with the screw group; *P < 0.05 compared with the screw group; ***P < 0.001 compared with the screw group; ****P < 0.0001 compared with the screw group

Patients in the screw group and the buttress group took longer to reach total weight than those in the joint group (*P* < 0.05) (Fig. 7a). Among all patients who completed internal fixation removal, the screw group had the longest internal fixation time (*P* < 0.05) (Fig. 7b). AOFAS scores (Fig. 7c) and VAS scores (Fig. 7d) of the screw group were evidently lower than those of the combined fixation group (all *P* < 0.05).

### Complex types of fractures

There were 16 patients with complex fractures complicated with articular surface collapse, of whom 6 had intraoperative allograft bone implants, 2 had delayed fracture union, and 2 had arthritic complications complicated with bone defects and bone grafting.

In terms of complications, the application of bulker-volume buttresses (3 cases) resulted in poorer wound healing than in the buttress group with vertical medial malleolus fractures alone (5 cases).

There were 8 complicated tibial fractures among patients with complex fractures, including 1 case of delayed healing (lower tibial fracture) and 1 case of wound infection (medial malleolus fracture), as shown in Table 2.

**Table 2:** Basic data and complication statistics of patients with special fractures buttress plate fixation

Index	Combined articular surface collapse (*n* = 16)	Combined lower tibial fracture (*n* = 8)
Gender (male/female)	11/5	5/3
Age	45.09 ± 6.89	48.75 ± 2.63
Combined fibula fracture (*n*)	10	4
Internal fixation failure (*n*)	1	0
Abnormal fracture growth and healing (*n*)	3	1
Combined with osteoarthritis (*n*)	3	1
Wound abnormality (*n*)	3	1
Full load time (week)	13.59 ± 0.87	13.35 ± 1.15
Internal fixed time (month)	13.18 ± 1.08	14.00 ± 1.41

After 2 years of postoperative follow-up, the AOFAS score and VAS score of the following two groups had good results: 91.18 ± 6.05 and 2.18 ± 1.08 in the patients with joint surface collapse, and 92.50 ± 4.80 and 2.50 ± 1.29 in patients with tibial fracture, with 100% excellent and good rate.

## Discussion

Medial malleolar fractures have been recognized as fractures around the ankle joint. Under special circumstances, the condition can progress to intra-articular fractures characterized by articular surface collapse, so good reduction and strong fixation are particularly important in medial malleolar fractures. To facilitate the diagnosis of these injuries and help guide treatment decisions, surgeons usually assess and classify ankle fractures according to the Herscovici classification system [15]. A large number of retrospective studies have shown that solitary medial malleolar fractures, surgically stable fibulas and well-reduced medial malleolar fractures, are not the indication for surgical treatment, but limited reports exist with regard to the vertical medial malleolar fractures that are extremely unstable [18]. Carter et al. reported that even supination-adduction type II (SAD-II) fracture configurations with medial malleolar vertical shear were excluded from such fractures [19]. Therefore, even if the reported nonsurgical treatment of isolated medial malleolar fractures yields a healing rate as high as 96% [5, 15], conservative treatment of this particular fracture type is not recommended.

Vertical medial malleolus fractures are a typical supination adduction injury, which are extremely unstable and usually require fixation [13]. The methods of internal fixation for medial malleolar fractures are complex and diverse, including screw fixation [20–23], tension band fixation and steel plate fixation [8, 12]. According to previous reports, the nonunion rate with single cortical lag screw fixation was 20% [21]. Wegner et al. showed that the anti-slip plate was stronger than double cortical screws, divergent monocortical screws and parallel monocortical screws with greater load capacity [24]. However, this conclusion still needs to be elucidated and confirmed in clinical practice. On the other hand, in a study involving 111 medial malleolar fractures by Ebraheim et al., 7 cases of vertical fractures were discussed, and except for 1 case using lag screw fixation, the rest of the cases achieved good results with support plates [7]. Due to the small number of vertical fracture cases in the study, the statistical analysis did not show any significant difference.

**Fig. 8:**
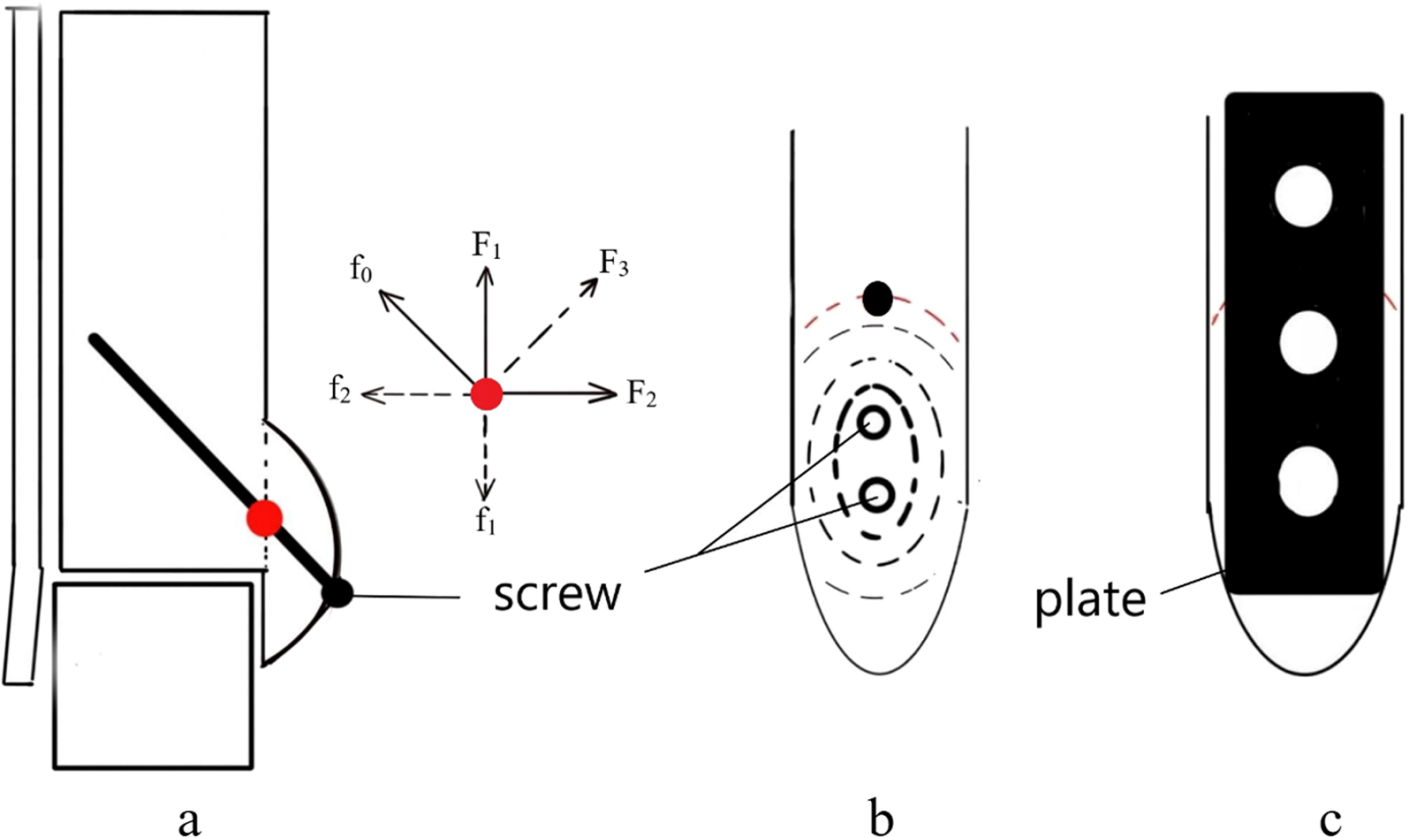
When the screw is used for fixation, the long axis of the screw (thick black line) has an intersection with the fracture line, which is marked as a red dot (**a**, medial malleolar front view). Here is the main force point of the screw. The main force analysis at this point is shown in the figures: *F*_1_ is the longitudinal shear force, *F*_2_ is the horizontal force, *F*_3_ is the resultant force of *F*_1_ and *F*_2_ (the direction is between *F*_1_ and *F*_2_), *f*_0_ is the active force of the long axis of the screw, *f*_1_ is the passive force of the short axis of the screw, and f_2_ is the component of the force of the screw. If the fixing effect of the screw is not enough to resist the external force of the fractured end during the movement, the fracture piece will collide with the direction of the black dot (**b**, side view of the medial malleolus) at the fracture line (red dashed line). The steel plate can provide comprehensive support by virtue of its larger coverage area (**c**, side view of the medial malleolus)

In the present study, it was found that the screw broke when encountering a large longitudinal shear force, which was not available in other fixing methods. Another screw-specific complication was that there was a small bone fragment at the proximal end of the fracture line that could be found in the long-term follow-up X-ray of the ankle joint after the operation, whereas it was not observed in the short-term postoperative X-ray. The causes of the above complications may be attribute to the fact that there is an “intersection point” at the intersection of the screw and the fracture line, where the main force on the fracture piece comes from the large vertical shear force F_1_ and the horizontal force during activity acting on *F*_2_, and the combined force of the two main forces allows the fractured end to have a tendency to move along the direction of *F*_3_. The active pressure of screw fixation is mainly *f*_0_ along its long axis. Although *f*_0_ can provide a part of the horizontal force against *F*_2_, *f*_1_ against *F*_1_ is passively provided by the thin short diameter of the screw (Fig. 8a). When the shear force increases, the screw is likely to bend and break, which may lead to fixation failure. The steel plate can be fully compressed based on the shape and characteristics of the device itself (Fig. 8c); on the other hand, it is because of the compression of the screw on the fracture surface. The effect is that the screw insertion point is gradually weakened to the surroundings. When the screw is not firmly fixed, the vertical shear force makes the bone block have an unstable tendency to move, and it is easy to hit the edge of the bone cortex at the proximal end of the fracture block during movement. There is formation of a small raised fragment (Fig. 8b). A wide volume of the steel plate and a full support force can completely prevent this from happening.

The two groups of cases using steel plate fixation showed a better internal fixation effect than the cases involving screws. However, it should be noted that 16 patients had different degrees of disuse osteoporosis that did not occur in patients with other fixation methods, more professional and detailed researches are therefore needed for the underlying reason. Nonetheless, these findings may be attributed to the use of a smaller buttress plate in the study to reduce the surgical incision and soft tissue dissection. The first screw at the distal end was anchored only at the tip of the medial malleolus, resulting in only one screw at the distal end of the fracture for effective fixation (Fig. 3c). The fixation was unstable, which may be one of the reasons for the failure of buttress steel plate fixation. There was no osteoporosis in the distal end of the combined application group because of screw-assisted fixation. In addition, unlike the high rate of poor wound healing in other studies, there was no statistically significant difference between the use of buttress plate fixation and screw fixation for wound recovery in patients with medial malleolar fractures in this study. Because the buttress plate used in this study was smaller, even the combined application of screws did not increase the surgical incision and soft tissue dissection.

A medial malleolar fracture can be an isolated fracture, or it can be a part of a double malleolar fracture or a triple malleolar fracture, hence it is important to select the best fixation method [25, 26]. Isolated vertical medial malleolar fractures are not the most common type in clinical practice, which only occur in approximately 7% of ankle fractures, but the incidence of combined lateral malleolar fractures is three times that of the former (20%). Therefore, while fixing a fracture of the medial malleolus, the fixation of the fracture of the lateral malleolus can also be a problem that needs to be considered, along with strong fixation of the fibula specifically for patients with fibula fractures. Studies are available to show that reliable support of the fibula is essential for maintaining the stability of medial malleolar fractures. Special attention is required for fractures that affect the articular surface, due to its proneness of occurrence of traumatic arthritis if not handled properly. In this study, the use of bone grafting and buttress plate fixation exerted good fixation effect. The buttress plates that can be used to fix vertical medial malleolus fractures have different shapes that show fundamental advantage of great toughness, perfectly fitting the bone surface, and being fixed by compression. Before the use of buttress plates for ankle joint fractures, there were reports confirming its application for fractures in the elbow joint coronoid process and trochlear fractures, ulnar carpal metacarpal joint fractures and dislocations, posterior sternoclavicular joint dislocations, and high-energy fractures of the lower limbs (such as tibial plateau posterior column fractures and spiral fractures of the tibia shaft) [27–29]. In this study, the buttress plate was also applied to fix both lower tibial fractures and vertical medial malleolar fractures. In the included 8 fracture cases, the fixation effect was good except for wound healing factors. The conventional use of steel plates to fix tibial fractures and separate fixation of medial malleolar fractures with screws may produce longer and more surgical incisions. However, the incisions were acceptable with the use of buttress plates in this study.

Of note, there were several limitations in this study. First, the number of patients in each subgroup was relatively small. Second, considering that the patients included in this study were from a single research center, multi-center studies with longer follow-ups are needed to confirm the current conclusions. Third, AOFAS score was used as an indicator of patient prognostic function evaluation. Although AOFAS score is one of the commonly used evaluation systems, the AOFAS society has recently recommended not to use it anymore.

## Conclusions

Based on our data, favorable fixation effect of buttress plate was found in patients with simple and complex vertical medial malleolus fractures. Despite the fact that this fixation method may cause poor wound healing and extensive soft tissue dissection, buttress plate fixation may provide a novel insight into medial malleolar fractures, especially for extremely unstable medial malleolar fractures.

## Data Availability

The datasets generated and analyzed during the current study are available from the corresponding author on reasonable request.

## References

[1] Egger AC, Berkowitz MJ (2018). Operative treatment of the malunited fibula fracture. Foot Ankle Int.

[2] Varenne Y, Curado J, Asloum Y, Salle de Chou E, Colin F, Gouin F (2016). Analysis of risk factors of the postoperative complications of surgical treatment of ankle fractures in the elderly: a series of 477 patients. Orthop Traumatol Surg Res.

[3] Lambert LA, Falconer L, Mason L (2020). Ankle stability in ankle fracture. J Clin Orthop Trauma.

[4] Carter TH, Duckworth AD, White TO (2019). Medial malleolar fractures: current treatment concepts. Bone Jt J.

[5] Wegner AM, Wolinsky PR, Robbins MA, Garcia TC, Maitra S, Amanatullah DF (2018). Headless compression screw fixation of vertical medial malleolus fractures is superior to unicortical screw fixation. Am J Orthop (Belle Mead NJ).

[6] Carney J, Ton A, Alluri RK, Grisdela P, Marecek GS (2020). Complications following operative treatment of supination-adduction type II (AO/OTA 44A2.3) ankle fractures. Injury.

[7] Ebraheim NA, Ludwig T, Weston JT, Carroll T, Liu J (2014). Comparison of surgical techniques of 111 medial malleolar fractures classified by fracture geometry. Foot Ankle Int.

[8] Blake S, Yakubek G, Shaer J (2015). Use of a locked fibular plate for fixation of a vertical shear medial malleolus fracture: a case report. J Foot Ankle Surg.

[9] Wegner AM, Wolinsky PR, Cheng RZ, Robbins MA, Garcia TC, Amanatullah DF (2017). Sled fixation for horizontal medial malleolus fractures. Clin Biomech (Bristol, Avon).

[10] Maniar H, Kempegowda H, Tawari AA, Rutter MR, Borade A, Cush G, Horwitz DS (2017). Medial malleoli fractures: clinical comparison between newly designed sled device and conventional screws. Foot Ankle Spec.

[11] Jones DA, Cannada LK, Bledsoe JG (2016). Are hook plates advantageous compared to antiglide plates for vertical shear malleolar fractures?. Am J Orthop (Belle Mead NJ).

[12] Uygur E, Poyanli O, Mutlu İ, Çelik T, Akpinar F (2018). Medial malleolus fractures: a biomechanical comparison of tension band wiring fixation methods. Orthop Traumatol Surg Res.

[13] Amanatullah DF, Khan SN, Curtiss S, Wolinsky PR (2012). Effect of divergent screw fixation in vertical medial malleolus fractures. J Trauma Acute Care Surg.

[14] Haller JM, Ross H, Jacobson K, Ou Z, Rothberg D, Githens M (2020). Supination adduction ankle fractures: ankle fracture or pilon variant?. Injury.

[15] Herscovici D, Scaduto JM, Infante A (2007). Conservative treatment of isolated fractures of the medial malleolus. J Bone Jt Surg Br.

[16] Van Lieshout EM, De Boer AS, Meuffels DE, Den Hoed PT, Van der Vlies CH, Tuinebreijer WE, Verhofstad MH (2017). American Orthopaedic Foot and Ankle Society (AOFAS) Ankle-Hindfoot Score: a study protocol for the translation and validation of the Dutch language version. BMJ Open.

[17] Sung YT, Wu JS (2018). The visual analogue scale for rating, ranking and paired-comparison (VAS-RRP): a new technique for psychological measurement. Behav Res Methods.

[18] Turhan E, Doral MN, Demirel M, Atay AO, Bozkurt M, Bilge O, Huri G, Atesok K, Kaya D (2013). Arthroscopy-assisted reduction versus open reduction in the fixation of medial malleolar fractures. Eur J Orthop Surg Traumatol.

[19] Carter TH, Oliver WM, Graham C, Duckworth AD, White TO (2019). Medial malleolus: Operative Or Non-operative (MOON) trial protocol - a prospective randomised controlled trial of operative versus non-operative management of associated medial malleolus fractures in unstable fractures of the ankle. Trials.

[20] Meeks BD, Kiskaddon EM, Boin MA, Willen B, Patel T, Prayson MJ (2020). The role of far cortical endosteal fixation for the treatment of medial malleolus fractures: a biomechanical study. J Foot Ankle Surg.

[21] Mandel J, Behery O, Narayanan R, Konda SR, Egol KA (2019). Single- vs 2-screw lag fixation of the medial malleolus in unstable ankle fractures. Foot Ankle Int.

[22] Bäcker HC, Konigsberg M, Freibott CE, Rosenwasser MP, Greisberg JK, Vosseller JT (2019). Radiographic results of unicortical medial malleolar fracture fixation. Foot Ankle Int.

[23] Bulut T, Gursoy M (2018). Isolated medial malleolus fractures: conventional techniques versus headless compression screw fixation. J Foot Ankle Surg.

[24] Wegner AM, Wolinsky PR, Robbins MA, Garcia TC, Maitra S, Amanatullah DF (2016). Antiglide plating of vertical medial malleolus fractures provides stiffer initial fixation than bicortical or unicortical screw fixation. Clin Biomech (Bristol, Avon).

[25] Bulut T, Gursoy M, Ertem H (2021). Fully threaded headless compression screw versus partially threaded cancellous lag screw in medial malleolus fractures: clinical and radiological outcomes. Eur J Trauma Emerg Surg.

[26] Barnes H, Cannada LK, Watson JT (2014). A clinical evaluation of alternative fixation techniques for medial malleolus fractures. Injury.

[27] Zhang K, Cui R, Gu Y, Wang D, Yan J, Yin Z, Xu C (2020). Posteroanterior lag screws versus posterior buttress plate fixation of posterior malleolar fragments in spiral tibial shaft fracture. J Foot Ankle Surg.

[28] Li J, Yin P, Zhang L, Chen H, Tang P (2019). Medial anatomical buttress plate in treating displaced femoral neck fracture a finite element analysis. Injury.

[29] Demir MT, Ertan Birsel S, Salih M, Pirinçci Y, Birsel O, Kesmezacar H (2020). Outcome after the surgical treatment of the Dubberley type B distal humeral capitellar and trochlear fractures with a buttress plate. Acta Orthop Traumatol Turc.

